# Laparoscopic Splenectomy: Postero-Lateral Approach

**Published:** 2019-01-12

**Authors:** A Garzi, G Ardimento, U Ferrentino, S Brongo, R.M Di Crescenzo, E Calabrò, M. S Rubino, E Clemente

**Affiliations:** 1 Division of Pediatric M.I.S. and Robotic Surgery University of Salerno, Italy; 2 Division of Pediatric Surgery University of Salerno, Italy; 3 Division of Plastic Surgery University of Salerno, Italy; 4 Department of Advanced Biomedical Sciences, Pathology Unit, University of Naples Federico II

**Keywords:** splenectomy in children, laparoscopic splenectomy, lateral approach

## Abstract

In paediatric population, the laparoscopic splenectomy has been preferred to the open surgery during the last years. Due to the improvement of the technique and the devices, the indications to the laparoscopic splenectomy have been increased, even though there is still a variety of conditions in which the execution of this technique is arduous. During the preoperative consult there is the need to carefully evaluate the existence of cholecystic lithiasis, the haemoglobin level in patients with SCA, platelet count in children with ITP and the vaccination status. An anterior and a lateral or hanging spleen approach are primarily used for laparoscopic splenectomy. In the last four years, near the Section of Pediatric Surgery of the Department of Pediatrics, Obstetrics and Medicine of the Reproduction of Siena University, 8 cases of splenomegaly have been treated, 7 by lateral videolaparoscopic splenectomy (5 males and 2 females, with medium age of 10,5 years) and 1 by anterior approach (10 years).

The advantages shown by these techniques allow the laparoscopic splenectomy to be considered as a valid alternative to the open surgery. In children’s laparoscopic splenectomy, the rate of complications is considerably low and the the major problem is the intraoperative hemorrhage. With increasing surgical experience, the minimally invasive approach appears to be superior in terms of faster postoperative recovery, shorter hospital stay, perioperative and postoperative advantages. Therefore, the laparoscopic technique may soon be accepted as the standard method in patients requiring splenectomy.

## I. INTRODUCTION

Splenectomy in children is a relatively common surgical procedure; for the paediatric population it is an important therapeutic option in many frequent conditions, especially in a variety of haematological and immunological disorders and in trauma (1). The first report describing laparoscopic splenectomy in adults was performed by Dalaitre et al in 1991, and subsequently in children by Tulman et al in 1993 (2,3). Development of advanced laparoscopic techniques and their most recent application to the paediatric patients allow the majority of splenectomies to be performed safely using minimally invasive techniques (1).

## II. INDICATIONS OF LAPAROSCOPIC SPLENECTOMY

Splenectomy in children is usually performed for several disorders (Tables 1, 2). Generally, concurrent laparoscopic cholecystectomy with splenectomy is recommended if stones are present. At first, the laparoscopic approach was highly contraindicated in small patients (< 10 Kg), including patients with significant splenomegaly, with previous abdominal surgeries and platelet counts less than 50.000. As a result of the improvements in technique and devices, however, laparoscopic splenectomy is nowadays possible in this category of patients. Conditions that may preclude an endoscopic approach are massive splenomegaly in a small infant or child (e.g. the spleen extends beyond the iliac crest); with limited manoeuvrability caused by the small abdominal cavity or hypersplenism secondary to portal hypertension considering that varices may be difficult to control (1).

## III. PREOPERATIVE EVALUATIONS

All patients undergoing laparoscopic splenectomy are typed and crossmatched for one unit of packed red blood cells. Children with sickle cell anemia (SCA) receive red blood cell transfusions to raise their haemoglobin level to 10 g/d. Children with idiopathic thrombocytopenic purpura (ITP) usually can achieve a platelet count of at least 50.000/mm with oral steroids. However, they occasionally will require intravenous immunoglobulin (IgG) or RH O (D) immunoglobin (in Rh-positive patients). Before splenectomy, all children receive vaccinations for Pneumococcal and Neisseria meningitidis. Haemophilus influenza type B now is part of the routine in childhood immunizations and usually is not required as an additional immunization (4). No specific imaging studies are necessary other than ultrasonography for gallstone evaluations (1). Routine perioperative antibiotics should be used. Splenic artery embolization may be considered in obese patients (5).

## IV. OPERATIVE TECHNIQUE

An anterior and a lateral or hanging spleen approach are primarily used for laparoscopic splenectomy. The advantage of the anterior technique is that the spleen remains attached and, therefore, static for the majority of the procedure. However, the main disadvantage is that it requires precise dissection in the splenic hilum without good visualization of the posterior aspect of the hilum or spleen, and deep or hidden vessels, may be inadvertently injured (1). With the lateral approach, thanks to the position of the patients on the operating table, the weight of the spleen facilitates the dissection. Thus, this approach allows a complete mobilization of the spleen before division of the hilar vessels. This approach simulates the standard open technique more closely (1).

The instrumentation is common for both procedures. The primary monitor is placed near the patient’s left shoulder, perpendicular to the surgeon. Light cords, camera cables, and insufflations tubing can be passed off the table together along the most cephalad portion of the field. A second monitor may help the assistant and technician see more easily but is mandatory only if cholecystectomy is to be performed as well. Standard 5 mm instruments and telescopes are adequate for most splenectomies in children. In smaller patients (< 10 Kg), 3 mm instruments can be used. A 12 mm port is need for the endoscopic linear stapler. Insufflation pressures for most children equivalent to that used in adults; however, occasionally lower pressures of 10 to 12 mmHg are necessary to prevent significant respiratory complications.

## V. SURGICAL TECHNIQUE: ANTERIOR APPROACH

**Fig. 1:**
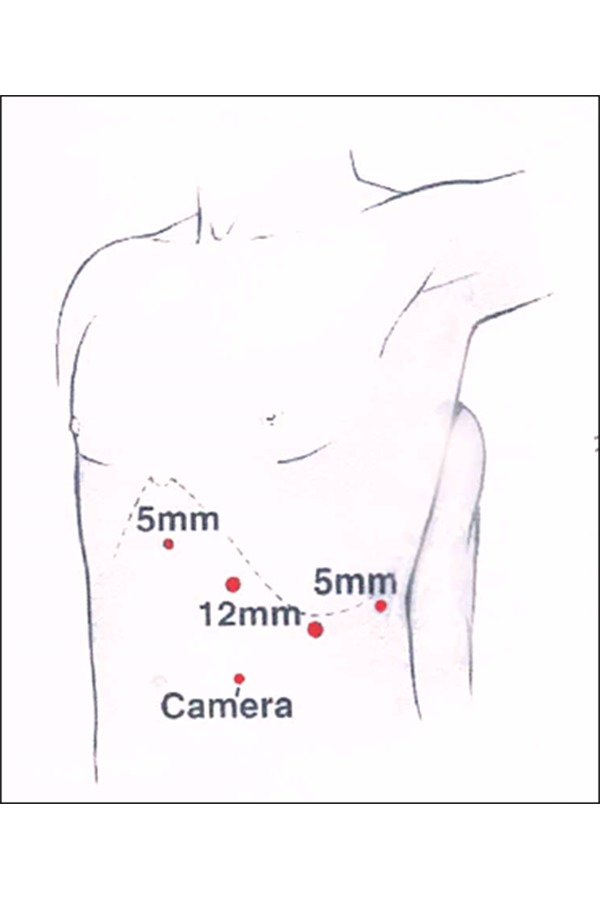
Typical port size and placement for laparoscopic splenectomy in a semilateral position.

**Fig. 2:**
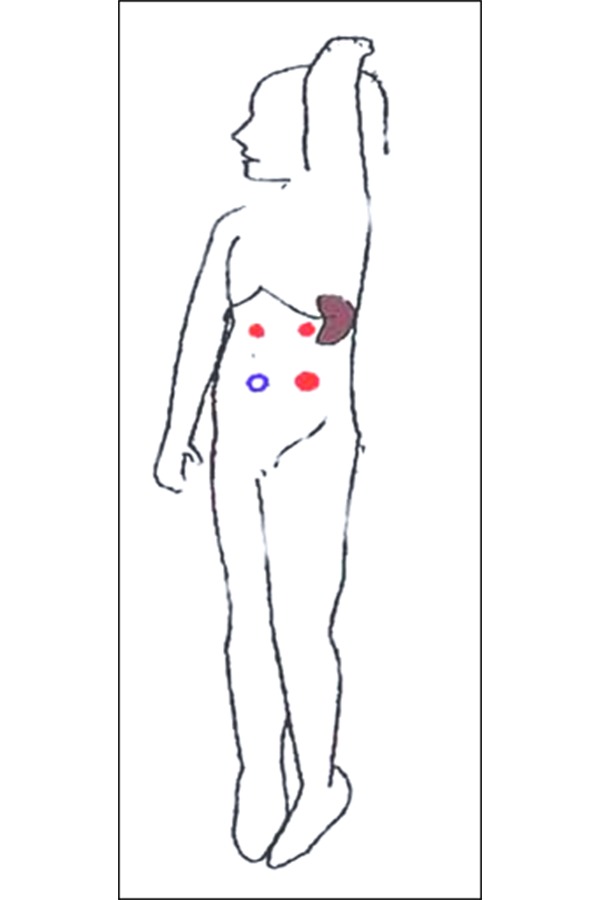
Position of the patient and ports for anterior approach

For the anterior approach, the patient is positioned supine with the surgeon standing on the patient’s right or in dorsal lithotomy (frog-legged for smaller children) with the surgeon at the foot of the table. Laparoscopic splenectomy can usually be completed with four ports as reported in figures 1–2. The spleen is exposed by downward traction on the colon and medial of the greater curvature of the stomach. Gravity holds the colon inferiorly. The lesser sac is entered through the gastrocolic ligament about the midpoint of the greater curvature. The branches of the left gastroepiploic and short gastric vessels are divided to completely separate the stomach and spleen. These vessels can be divided between clips but cautery or ultrasonic harmonic scalpel is quicker and equally secure. A fan retractor inserted through the left upper quadrant port is used to elevate the stomach and the lesser sac.

## VI. SURGICAL TECHNIQUE: LATERAL APPROACH

**Fig. 3:**
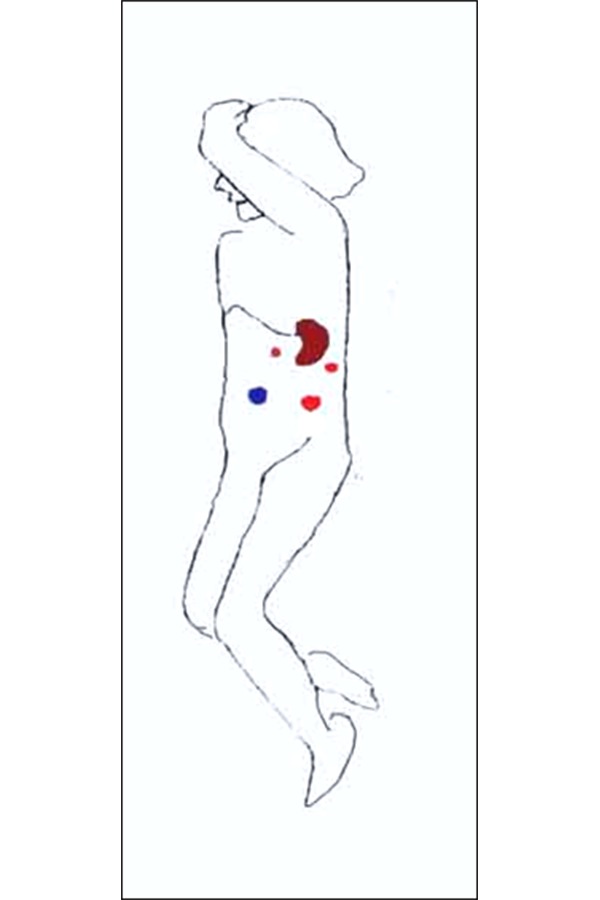
Position of the patient and ports for lateral approach

For the lateral approach, the patient is positioned in a full right lateral decubitus position. The table can be rotated 45° in either direction, and therefore the patient can be positioned as needed in a near-supine or full lateral position (Figure 3). In this approach, the exposure of the hilum is excellent because the stomach and colon fall away by gravity and require no retraction (6, 7). The peritoneal attachment, the splenorenal ligament and splenocolic ligament are divided leaving the organ suspended by only a 1 cm cuff of lateral peritoneal reflection. Through a 5 mm lumbar port the peritoneum is grasped to retract the spleen medially. Dissection of the splenic hilum is approached from the lower pole and continued in a cephalad progression. When these vessels are divided, the spleen can be manipulated into the specimen bag.

Many surgeons use to extract the spleen with endoscopic bags after morcellation procedure; although there is a number of endoscopic bags, most of them lacks the structural integrity withstand morcellation and rupture of the splenic contents into the peritoneal cavity can result in diffuse splenosis. For this reason in many centre, surgeons use to perform a Pfannenstiel’s incision for extracting the spleen. This procedure avoids the risk of a diffuse splenosis and it gives a good esthetic results.

## VII. POSTOPERATIVE CARE

Most patients tolerate the procedure extremely well. Intravenous morphine is used in the initial postoperative period, but few patients have required more than oral analgesics after the first 6 to 12 hours. A post-hospitalization visit is scheduled at about a week (1). Daily oral penicillin prophylaxis is recommended. The patients and his family should be aware of the concerns regarding post-splenectomy sepsis. Risk in patients with non-malignant hematological conditions is 2% or less. Death rate for affected patients is 2–10% (5).

## VIII. COMPLICATIONS

Complications of laparoscopic splenectomy can be grouped into those related to technical problems at the time of the procedure, those related to the patient’s physiologic status, or those that develop as a result of an underlying disease process, such as a sickle cell disease. Most paediatric series have documented a very low complication rate. In a review of 344 laparoscopy splenectomy reported between 1995 and 2002, a rate of 2.9% of Clavien grade I complications (minor complications that resolve spontaneously) and a 6.7% rate of grade II complications (potentially life-threatening, usually requiring intervention, iatrogenic injuries, or complications resulting in the doubling of the hospital stay) has been reported (4, 8). The greatest potential problem is hemorrhage, which can derive from three sources: a small caliber vessel (short gastric or polar vessels), a larger vessel of the hilum, or the splenic parenchyma. The first type of hemorrhage, although not life-threatening, can become quite a hindrance to the operations, as rapidly accumulating blood may impair vision. This hemorrhage, however, can also be easily stopped with the use of clips, electrocoagulation, or the ultrasonic dissector. Hemorrhage from a larger vessel may be an indication for immediate conversion to laparotomy. Another potential complication of laparoscopic splenectomy is injury to the tail of the pancreas. Proper dissection and placement of the endostapler can avoid this problem. Other complications reported include deep vein thrombosis, pulmonary embolus, and wound infection (9).

Conversion of laparoscopic splenectomy to the open procedure is not uncommon, especially during the initial part of a surgeon’s experience. Generally, the reason for conversion has been hemorrhage not readily amenable to laparoscopic control and requiring immediate legation of a large vessel. One should not hesitate to abort the laparoscopic approach if the anatomy is unfavorable or the patient’s condition is jeopardized by hemorrhage. An alternative method in these cases could be hand-assisted laparoscopic splenectomy, with the advantage of immediate vessel control by finger pressure (7).

In the literature, the incidence of accessory spleens has been reported between 10 and 40% of patients (1,9) and it can be found in the splenic fossa next to the colon or stomach, as well as in the upper omentum or below the mesocolon (9).

## IX. PERSONAL EXPERIENCE

In the last four years, near the Section of Pediatric Surgery of the Department of Pediatrics, Obstetrics and Medicine of the Reproduction of Siena University, 8 cases of splenomegaly have been treated, 7 by lateral videolaparoscopic splenectomy (5 males and 2 females, with medium age of 10,5 years) and 1 by anterior approach (10 years); 5 cases of hereditary spherocytosys, 1 thrombocytopenia and 1 infect epidermoid cysts; in 3 cases cholecystectomy with laparoscopic technique has been associated; in 1 case it has been necessary to convert via open. In the males, spleen extraction has been carried out through an inguinal access, while in the females by means of minium-laparotomy of Pfanniestiel. The medium dimension of the milza has been 14X9 cm with a range from (14–19) X (7–11) cm. The operative time averaged 187 minutes (interval 150–240 minutes). The average stay has been 4.7 days with the lateral approach as compared with 6 days for then anterior approach.

## X. CONCLUSION

The minimally invasive approach, compared with open splenectomy, requires more operating room time but appears to be superior in terms of faster postoperative recovery, shorter hospital stay, lower perioperative morbidity and duration of postoperative analgesia. With increasing surgical experience, shorter operative time, the postoperative advantages of laparoscopic, versus open surgery, and decreasing cost, laparoscopic splenectomy is emerging as the procedure of choice and soon may be accepted as the standard method in patients requiring splenectomy, especially for immune thrombocytopenic purpura and hereditary spherocytosis.

## Figures and Tables

**Table 1:** Laparoscopic splenectomy: indications.

Indications of laparoscopic splenectomy
All haemolytic disorders
Immune purpura (ITP)
AIDS related thrombocytopenic
Hodgkin’s and non-Hodgkin’s diseases
Splenic cyst
Primary and secondary hypersplenism
Gaucher’s disease
Ectopic spleen
Sarcoidosis

**Table 2:** Laparoscopic splenectomy: relative contraindications

Relative contraindications of laparoscopic splenectomy
High risk for general anaesthesia
Coagulopathy
Massive splenomegaly
Portal hypertension with bleeding oesophageal varix (Hassab’s technique)
Splenic abscess
Spleen artery aneurysm
Accidental for iatrogenic rupture
Post-traumatic rupture
